# Vaccine Matter from the Cow

**Published:** 1866-01

**Authors:** 


### Miscellaneous.

### Vaccine Matter from the Cow.

It will be recollected that at the Medical Congress at Lyons last year, Professor Palasciano, of Naples, gave an interesting account of the practice of vaccinating from the cow followed at Naples for the last sixty years. At that time a kind of panic prevailed in France respecting transmission of syphilis through the medium of vaccine lymph, and the Neapolitan plan was warmly welcomed as offering an effectual preventive to such a diaster. Cows were accordingly vaccinated in Lyon and Paris; but as we have heard nothing whatever of late of the plan being pursued, we suppose that either practical difficulties or a suspicion that great exaggeration had prevailed concerning the evils attendant upon the ordinary mode of vaccination have prevented its being continued.—However this may be, the Prussian Government, having had its attention drawn to the matter, has caused a report to be prepared, of which Dr. Müller gives an account in a recent number of the *Berlin Medical Wochenschrift.* It seems that a Dr. Galbiati commenced the practice of vaccinating directly from the cow at Naples sixty years ago, and that it has been regularly continued from that time by Signors 'Feola and Negri. From the years 1849 to 1859, the cows employed were the property of the King, who had his own family vaccinated in this manner; but since then Negri has conducted the vaccinations as a private speculation, charging five francs for bringing the cow and vaccinating the child at its parents' residence. The generation of the vaccine pustules does not seem to injure the cow, but the udders, upon which are often from 80 to 100 pustules, are so much damaged that the animal is usually slaughtered, the price obtained for it going towards covering the expenses. It is remarkable, however, that this mode of vaccination does not seem to have become, even after so loug a period, very popular in Naples, although warmly supported by influential persons, the ordinary vaccinations being performed just as in other countries, the employment of Negi's cows being confined to the richer persons and the re-vaccination of a portion of the army.

Some of the Neapolitan lymph having been forwarded to Berlin,

Dr. Müller made some comparative experiments with it and with lymph derived from other sources, and found the results identical. He also vaccinated some cows and calves with the lymph, generally producing true vaccine pustules, which attained their full development by the sixth or seventh day. They were, however, far from containing the quantity of lymph stated by Negri to be produced, this not running out after the separation of the scab, as is usually the case in the human pustule, so that the glass tubes were filled only with difficulty. This lymph produced true pustules in some children, while in others it did not succeed. The milk of the cows submitted to the process diminished in quantity, and the animals became emaciated. Alarmed also, they offered much resistance to both the inoculation and abstraction of the lymph; so that it is evident that Negri's cows must furnish a larger supply of lymph, and belong to a quieter race, or he must be much more expert in their management to enable him to take them to the patients' houses for the small sum of five francs. However, the trials made establish the possibility of vaccinating direct from the cow in all cases, but they also show that it is neither as easy or cheap process. After all, is there any great advantage in substi tuting this mode of proceeding for that from arm-to-arm vaccination? and is there not at least as much danger of transplanting diseases of the cow as of conveying syphilitic poison, especially as the best and most robust cows would naturally not be those employed for this purpose?—*Med. Times and Gaz.* Oct. 28, 1865. *Medical News.*

